# Analysis of Temporal Trends and Variation in the Use of Defibrillation Testing in Contemporary Practice

**DOI:** 10.1001/jamanetworkopen.2019.13553

**Published:** 2019-10-18

**Authors:** Ryan T. Borne, Tiffany Randolph, Yongfei Wang, Jeptha P. Curtis, Pamela N. Peterson, Frederick A. Masoudi, Amneet Sandhu, Matthew M. Zipse, Kevin Thomas, Valentina Kutyifa, Nihar R. Desai, Yong-Mei Cha, Jonathan C. Hsu, Andrea M. Russo

**Affiliations:** 1 Division of Cardiology, University of Colorado Anschutz Medical Campus, Aurora, Colorado; 2 Cone Health Medical Group HeartCare, Greensboro, North Carolina; 3 Department of Medicine, Yale University School of Medicine, New Haven, Connecticut; 4 Center of Outcomes and Research Evaluation, Yale-New Haven Health, New Haven, Connecticut; 5 Department of Medicine, Denver Health Hospital, Denver, Colorado; 6 Department of Medicine, Duke University School of Medicine, Durham, North Carolina; 7 Department of Medicine, Rochester Hospital, Rochester, New York; 8 Department of Medicine, Mayo Clinic, Rochester, Minnesota; 9 Department of Medicine, University of California, San Diego, La Jolla; 10 Department of Medicine, Cooper Medical School of Rowan University, Camden, New Jersey

## Abstract

**Importance:**

Defibrillation testing (DFT) is performed during implantable cardioverter-defibrillator (ICD) implantation to assess the capacity of the device to detect and terminate ventricular arrhythmias. However, DFT can result in complications and omission of its use has been shown to be safe.

**Objective:**

To describe temporal trends and variation in the use of DFT in contemporary practice in the United States.

**Design, Setting, and Participants:**

This multicenter cross-sectional study used data from the National Cardiovascular Data Registry ICD Registry. A total of 499 211 patients from 1794 different facilities undergoing first-time ICD implantation from April 2010 to December 2015 were included. Data analysis was performed from May 20, 2015, to August 15, 2019.

**Exposure:**

Defibrillation testing was assessed using the National Cardiovascular Data Registry ICD Registry.

**Main Outcomes and Measures:**

Defibrillation testing rates and median odds ratios (MORs) were assessed over time. The MOR represents the odds that a randomly selected patient receiving testing at a hospital with high testing rates would be tested compared with if he or she had received care at a hospital with low testing rates.

**Results:**

Of the 499 211 patients from 1794 different facilities included in this analysis, the mean (SD) age of the population was 65.5 (13.4) years and 356 681 patients (71.4%) were men. The use of DFT declined from 71.6% in the first calendar quarter of 2010 to 36.4% in the fourth quarter of 2015 (*P* < .001). Patients undergoing DFT were more likely than those without testing to have ischemic heart disease (170 569 [58.1%] vs 116 295 [56.6%]), ventricular tachycardia (91 500 [31.2%] vs 58 949 [28.7%]), and less advanced heart failure (New York Heart Association class I and II, 153 188 [52.2%] vs 91 215 [44.4%]) (*P* < .001 for all). The MOR for the use of defibrillation testing was 3.78 (95% CI, 3.54-4.03) in 2010, increasing to 6.05 (95% CI, 5.61-6.52) in 2015, indicating that by 2015 a randomly selected patient receiving testing at a hospital with high testing rates would have a 6-fold higher odds of being tested than if they had received care at a hospital with low testing rates.

**Conclusions and Relevance:**

Defibrillation testing at the time of ICD placement in the United States may have declined over time; however, institutional variation in its use appears to be marked and increased. This variability in the reduced use of defibrillation testing could reflect differences in individual or institutional cultures of practice.

## Key Points

**Question:**

What are contemporary trends, institutional variation, and patient and hospital characteristics associated with defibrillation testing among patients undergoing first-time implantable cardioverter-defibrillator implantation?

**Findings:**

In this cross-sectional study of 499 211 patients undergoing implantable cardioverter-defibrillator implantation, there was a significant decline in the use of defibrillation testing, yet there was a marked increase in institutional variation.

**Meaning:**

There is variability in the deadoption of defibrillation testing which is not based on patient characteristics and could reflect differences in individual or institutional practices.

## Teaser

This cross-sectional study examines temporal trends and variations in the use of defibrillation testing among patients undergoing first-time implantable cardioverter-defibrillator implantation.

## Introduction

Implantable cardioverter-defibrillators (ICDs) improve the survival of patients at high risk for sudden death.^1,2,3,4,5^ Defibrillation testing (DFT) provides a means to evaluate the ability of a newly implanted device to detect and terminate ventricular fibrillation. Defibrillation testing was routinely used in the randomized clinical trials for which they are indicated, is included in the US Food and Drug Administration’s labeling of ICDs, and the testing was adopted in routine clinical practice.^6,7^

However, the benefits of DFT have not routinely been demonstrated. Several studies published between 2008 and 2012 found no association between routine testing and the efficacy of ICD shocks during follow-up or the risk of arrhythmic death.^8,9,10,11^ Furthermore, contemporary ICDs rarely fail in their ability to detect and treat ventricular arrhythmias. Improvements in device technology provide defibrillation thresholds that are typically 10 to 20 J less than the maximum ICD output such that successful defibrillation occurs in more than 90% of clinical shocks.^6,10,11,12,13,14,15,16^ Complications of DFT, while relatively rare, can be serious.^11,12,13,14^

Given the evolution of the evidence and guidelines, we sought to evaluate contemporary temporal trends and institutional variation in DFT at the time of initial ICD implantation and determine patient and hospital characteristics associated with DFT within the National Cardiovascular Data Registry (NCDR) ICD Registry between 2010 and 2015. The results could provide a perspective on the deadoption of a widely used clinical practice.

## Methods

### Data Source

Details of the NCDR ICD Registry have been reported previously.^17,18,19^ The Centers for Medicare & Medicaid Services mandated that data on all Medicare primary prevention implants be entered into the NCDR ICD Registry until the data collection requirement ended on February 15, 2018.^17^ The mandate was in effect during the study years for this analysis. The registry uses both a standardized data set and definitions, has requirements in place to ensure uniform data entry and transmission, and is subject to data quality checks. All data submissions are evaluated for errors and completeness. This information is summarized in an automated report that is sent to the participants after each data submission. The NCDR audit program, which includes hospital medical records reviews and blinded data abstractions, serves as an additional mechanism to assess the accuracy of the data and enables participants to identify areas for improved data entry. Use of the NCDR was approved by the ICD Registry Research and Publications Committee. Statistical analysis was approved by the Yale Center for Outcomes Research and Evaluation. The initial analysis was performed on May 20, 2015. Research involving the NCDR is covered under a waiver of informed consent. This study followed the Strengthening the Reporting of Observational Studies in Epidemiology (STROBE) reporting guideline for cross-sectional studies.

### Study Population

All patients undergoing initial implantation of a transvenous primary or secondary prevention ICD (single chamber, dual chamber, or cardiac resynchronization with defibrillator) between April 2010 and December 2015 were included. Patients were excluded if their ICD procedure was a replacement of a prior system, a generator replacement, or a lead revision. Patients younger than 18 years were included but represented only 0.35% of the cohort. Patients receiving a nontransvenous ICD system or a subcutaneous ICD, as well as those without documentation about whether DFT was performed, were also excluded.

### Outcomes

Defibrillation testing was ascertained through documentation as described in the ICD Registry V2.1. In-hospital adverse events (cardiac arrest, cardiac perforation, lead dislodgement, myocardial infarction, cardiac tamponade, peripheral embolus, transient ischemia attack or cerebrovascular accident, urgent cardiac surgery) and in-hospital death were identified from the NCDR ICD registry.

### Statistical Analysis

Baseline characteristics were compared between patients who did and did not have DFT performed at the time of implantation using χ^2^ tests for categorical variables and 2-tailed, unpaired *t* tests for continuous variables. Continuous variables are presented as means (SDs) and categorical variables as frequency and percentage. Patient, hospital, and clinician factors were compared using the χ^2^ test for categorical variables and *t* test for continuous variables. Missing values were rare (≤1%) for all of the factors under consideration except left-ventricular ejection fraction (1.5%) and QRS interval duration (milliseconds) (7.1%); dummy variables indicating missing values for these factors were created and included in the models; missing values were imputed as the most frequent value of the categorical variables and the median of the continuous variables in the models. A multivariable analysis was performed to identify the patient and hospital factors associated with the use of DFT. Variables in the model were selected based on clinical experience and prior literature and included patient demographics, clinical characteristics, physician training, and hospital characteristics. Complications and death were adjusted using similar variables.

To assess temporal changes in DFT, we examined its use since 2010 by quarters and tested the trend using the Cochran-Armitage trend test. Subgroup analysis was then performed to assess trends in specific patient populations, including primary and secondary prevention indication, and patients with structural abnormalities, including hypertrophic cardiomyopathy and congenital heart disease. We also assessed use in patients according to their estimated risk for an elevated defibrillation threshold based on a risk score previously derived and validated in the NCDR ICD Registry.^20^ This score is calculated based on the following characteristics: age younger than 70 years (1 point), male sex (1 point), black race (4 points), Hispanic ethnicity (2 points), other race/ethnicity (1 point), New York Heart Association (NYHA) functional class III (1 point) or IV (3 points), no ischemic heart disease (2 points), renal dialysis (3 points), secondary prevention indication (1 point), and single-chamber (2 points) or biventricular (1 point) ICD type. A risk score of 7 or greater was used to identify patients at high risk for defibrillation safety margin. Temporal changes in DFT among subgroups was performed using the Cochran-Mantel-Haenszel test to examine the association between categorical variables (use of DFT among different years) controlling for the hospital use of DFT in quartiles.

Hospital variation in the use of DFT was assessed by stratifying hospitals into quartiles of all DFT across the study years. To assess the institutional level variation in use of DFT, we determined the distribution and interquartile ranges of DFT in the entire cohort and in each year. To quantify the extent to which variation was explained by hospital level effects, the hospital-specific median odds ratio (MOR) was calculated using a validated method for the entire cohort and for each year.^21^ Hierarchical logistic regression models were used to determine the between-hospital variance of DFT use for clustering of patients within hospital performed and the MOR was calculated. The MOR represents the odds that a randomly selected patient receiving DFT at a hospital with high testing rates would be tested than if he or she had received care at a hospital with low DFT rates. Findings were considered significant at *P* < .05. Data analysis was performed from May 20, 2015, to August 15, 2019, using SAS, version 9.4 (SAS Institute Inc).

## Results

### Study Population

**Figure 1.**
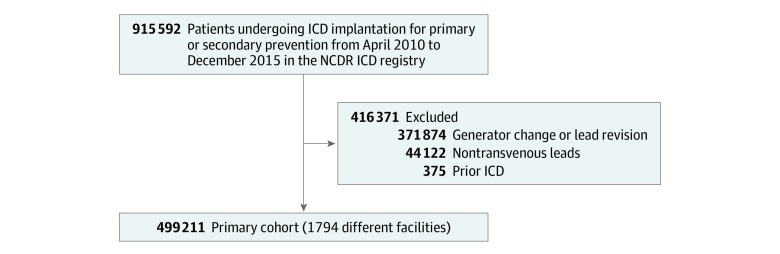
Attrition Plot ICD indicates implantable cardioverter-defibrillator; NCDR, National Cardiovascular Data Registry.

A total of 499 211 patients from 1794 different facilities were included in this analysis; the mean (SD) age of the population was 65.5 (13.4) years and 356 681 patients (71.4%) were men. The baseline demographics and characteristics based on DFT are included in the Table. Within the NCDR ICD registry, 915 592 patients underwent ICD implantation between April 2010 and December 2015 (Figure 1). There were 416 381 patients excluded from our final analysis owing to nontransvenous lead implantation (44 122), generator change or lead revision (371 874), prior ICD (375), and unknown use of DFT (10).

**Table.** Baseline Patient, Clinician, and Hospital Characteristics Stratified by DFT

Description	No. (%)		
	Total (N = 499 211)	DFT (n = 293 660)	No DFT (n = 205 551)
Demographics			
Age, mean (SD), y	65.5 (13.4)	64.7 (13.4)	66.6 (13.3)
Men	356 681 (71.4)	209 344 (71.3)	147 337 (71.7)
Race/ethnicity			
White and non-Hispanic	379 883 (76.1)	225 922 (76.9)	153 961 (74.9)
Black and non-Hispanic	76 509 (15.3)	44 099 (15.0)	32 410 (15.8)
Hispanic	30 461 (6.1)	16 754 (5.7)	13 707 (6.7)
Insurance payer			
Medicare	301 717 (60.4)	172 944 (58.9)	128 773 (62.6)
Medicaid	34 441 (6.9)	19 839 (6.8)	14 602 (7.1)
Private	136 233 (27.3)	84 416 (28.7)	51 817 (25.2)
History and risk factors			
Body mass index, mean (SD)^a^	30.0 (13.2)	30.0 (12.8)	30.0 (13.8)
Heart failure	405 868 (81.3)	231 308 (78.8)	174 560 (84.9)
NYHA functional classification			
Class I	68 829 (13.8)	46 131 (15.7)	22 698 (11.0)
Class II	175 574 (35.2)	107 057 (36.5)	68 517 (33.3)
Class III	238 521 (47.8)	132 601 (45.2)	105 920 (51.5)
Class IV	14 315 (2.9)	6685 (2.3)	7630 (3.7)
Nonischemic dilated cardiomyopathy	191 489 (38.4)	107 538 (36.6)	83 951 (40.8)
Syncope	84 259 (16.9)	51 988 (17.7)	32 271 (15.7)
Atrial fibrillation/flutter	161 299 (32.3)	78 510 (26.7)	82 789 (40.3)
Ventricular tachycardia	150 449 (30.1)	91 500 (31.2)	58 949 (28.7)
Cardiac arrest	56 294 (11.3)	34 784 (11.8)	21 510 (10.5)
Ischemic heart disease	286 864 (57.5)	170 569 (58.1)	116 295 (56.6)
Prior MI	239 218 (47.9)	143 316 (48.8)	95 902 (46.7)
Primary valvular heart disease	59 617 (11.9)	32 285 (11.0)	27 332 (13.3)
Cerebrovascular disease	74 556 (14.9)	40 849 (13.9)	33 707 (16.4)
Chronic lung disease	106 730 (21.4)	61 810 (21.0)	44 920 (21.9)
Diabetes	193 995 (38.9)	112 638 (38.4)	81 357 (39.6)
Sleep apnea	68 319 (13.7)	39 065 (13.3)	29 254 (14.2)
Currently receiving dialysis	14 884 (3.0)	8295 (2.8)	6589 (3.2)
Hypertension	400 683 (80.3)	234 351 (79.8)	166 332 (80.9)
Diagnostic studies			
Most recent LVEF, mean (SD), %	28.9 (11.8)	30 (12.1)	28 (11.2)
QRS interval duration, mean (SD), ms	122 (31.0)	120 (30.6)	123 (31.5)
Bundle-branch block			
Left	127 242 (25.5)	72 138 (24.6)	55 104 (26.8)
Right	50 271 (10.1)	28 682 (9.8)	21 589 (10.5)
Ventricular-paced rhythm	23 365 (4.7)	11 409 (3.9)	11 956 (5.8)
Creatinine, mean (SD), mg/dL	1.3 (1.1)	1.3 (1.1)	1.3 (1.1)
Sodium, mean (SD), mEq/L	139 (4.8)	139 (4.7)	138 (5.0)
Potassium, mean (SD), mEq/L	4.2 (0.5)	4.2 (0.5)	4.2 (0.5)
Brain-type natriuretic peptide, mean (SD), pg/mL	980 (1321.9)	889 (1237.4)	1098 (1415.9)
Procedure information			
ICD indication: primary prevention	397 514 (79.6)	228 847 (77.9)	168 667 (82.1)
Final device type			
Single chamber	138 218 (27.7)	78 097 (26.6)	60 121 (29.2)
Dual chamber	180 318 (36.1)	117 626 (40.1)	62 692 (30.5)
CRT-D	179 679 (36.0)	97 368 (33.2)	82 311 (40.0)
Medications			
ACE inhibitor or ARB	375 408 (75.2)	222 879 (75.9)	152 529 (74.2)
β-blocker	446 280 (89.4)	262 784 (89.5)	183 496 (89.3)
Diuretic	309 886 (62.1)	174 668 (59.5)	135 218 (65.8)
Antiarrhythmic agent	81 087 (16.2)	47 476 (16.2)	33 611 (16.4)
Anticoagulant therapy (warfarin)	124 622 (25.0)	59 367 (20.2)	65 255 (31.7)
Aspirin	351 123 (70.3)	211 459 (72.0)	139 664 (67.9)
Platelet aggregation inhibitors	123 088 (24.7)	76 200 (25.9)	46 888 (22.8)
Physician training			
Board-certified EP	349 749 (70.1)	206 364 (70.3)	143 385 (69.8)
EP fellowship only	54 646 (10.9)	30 212 (10.3)	24 434 (11.9)
Surgery boards	6219 (1.2)	3494 (1.2)	2725 (1.3)
Pediatric cardiology boards	589 (0.1)	314 (0.1)	275 (0.1)
HRS guidelines^b^	48 054 (9.6)	30 159 (10.3)	17 895 (8.7)
None of the above	36 104 (7.2)	21 096 (7.2)	15 008 (7.3)
Hospital characteristics			
Owner			
Government	6804 (1.4)	4340 (1.5)	2464 (1.2)
Private/community	419 519 (84.0)	252 515 (86.0)	167 004 (81.2)
University	72 888 (14.6)	36 805 (12.5)	36 083 (17.6)
Teaching	260 294 (52.1)	147 044 (50.1)	113 250 (55.1)
Public	246 858 (49.4)	145 917 (49.7)	100 941 (49.1)
Region			
Midwest	121 319 (24.3)	73 663 (25.1)	47 656 (23.2)
Northeast	92 137 (18.5)	47 722 (16.3)	44 415 (21.6)
South	213 133 (42.7)	130 159 (44.3)	82 974 (40.4)
West	72 382 (14.5)	41 982 (14.3)	30 400 (14.8)

Abbreviations: ACE, angiotensin-converting enzyme; ARB, angiotensin receptor blocker; CRT-D, cardiac resynchronization therapy with defibrillator; DFT, defibrillation testing; EP, electrophysiologist; HRS, Heart Rhythm Society; ICD, implantable cardioverter-defibrillator; LVEF, left-ventricular ejection fraction; MI, myocardial infarction; NYHA, New York Heart Association.

SI conversion factors: To convert brain-type natriuretic protein to nanograms per liter, multiply by 1; creatinine to micromoles per liter, multiply by 88.4; potassium to millimoles per liter, multiply by 1; sodium to millimoles per liter, multiply by 1.

^a^ Calculated as weight in kilograms divided by height in meters squared.

^b^ The HRS guidelines refer to an alternative pathway for non-electrophysiology-trained physicians to be credentialed to perform ICD implantation.

Most patients had ischemic heart disease (286 864 [57.5%]), categorized as NYHA class II to III heart failure (414 095 [82.9%]). The mean (SD) left-ventricular ejection fraction was 28.9% (11.8%). Most patients received a device for primary prevention of sudden death (397 514 [79.6%]).

### Defibrillation Testing

**Figure 2.**
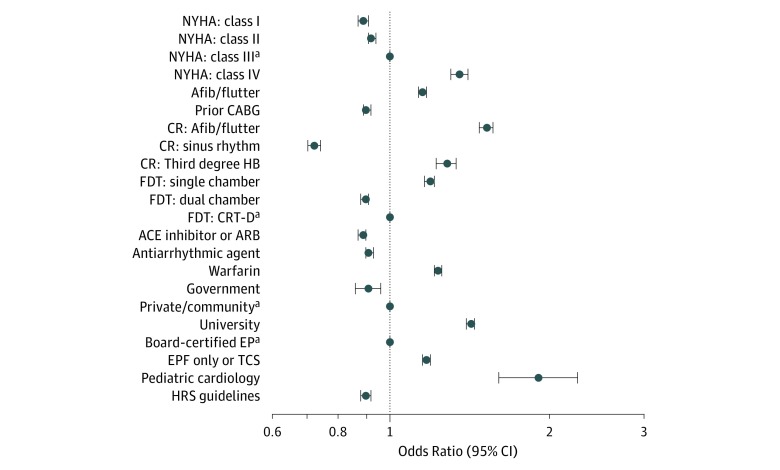
Factors Associated With Not Performing Defibrillation Testing ACE indicates angiotensin-converting enzyme; Afib, atrial fibrillation; ARB, angiotensin receptor blocker; CABG, coronary artery bypass graft; CR, cardiac resynchronization; CRT-D, cardiac resynchronization therapy with defibrillator; EP, electrophysiologist; EPF, electrophysiology fellowship; FDT, final device type; HB, heart block; HRS, Heart Rhythm Society; NYHA, New York Heart Association; and TCS, thoracic cardiac surgery. ^a^Variable with more than 2 categories.

Defibrillation testing was performed in 293 660 patients (58.8%) of the entire population, of which lowest energy testing was performed in 288 841 patients (57.8%), upper limit of vulnerability in 14 741 patients (3.0%), and both methods in 9592 patients (1.9%). Patients who underwent DFT were more likely than those who did not undergo DFT to have ischemic heart disease (170 569 [58.1%] vs 116 295 [56.6%]), prior ventricular tachycardia (91 500 [31.2%] vs 58 949 [28.7%]), syncope (51 988 [17.7%] vs 32 271 [15.7%]), cardiac arrest (34 784 [11.8%] vs 21 510 [10.5%]), and less advanced NYHA class I and II heart failure (153 188 [52.2%] vs 91 215 [44.4%]) (*P* < .001 for all) (Table). Possible indicators of not performing DFT are described in Figure 2. There was significantly lower use of DFT among patients who had NYHA class IV (odds ratio [OR], 1.35; 95% CI, 1.30-1.40) and those with atrial arrhythmias (OR, 1.52; 95% CI, 1.47-1.56) and higher use in patients with NYHA class I categorization (OR, 0.89; 95% CI, 0.87-0.91), sinus rhythm (OR, 0.72; 95% CI, 0.70-0.74), and antiarrhythmic agent therapy (OR, 0.91; 95% CI, 0.90-0.93). There were modest but statistically significant differences in the certification of clinicians performing the implantation and use of DFT at the time of initial ICD implantation: board-certified electrophysiologist (OR, 1.00; 95% CI, 1.00-1.00); electrophysiologist fellowship or thoracic-cardiac surgery (OR,1.17; 95% CI, 1.15-1.19); pediatric cardiology (OR, 1.90; 95% CI, 1.60-2.25); and HRS guidelines (OR, 0.90; 95% CI, 0.88-0.92). Patients undergoing implantation in university settings were less likely to have DFT (OR, 1.42; 95% CI, 1.39-1.44)

### Temporal Trends and Variation in DFT

**Figure 3.**
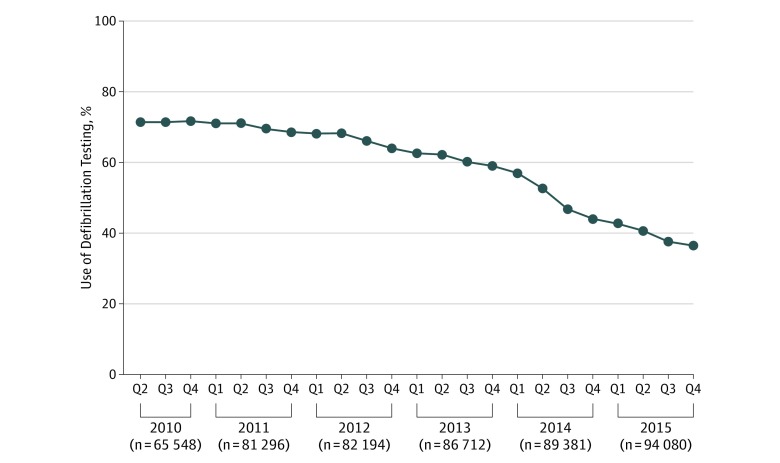
Temporal Trends in Defibrillation Testing The percentage of use of defibrillation testing among all hospitals per quarter (Q) from April 2010 to December 2015.

Temporal trends in the use of DFT are depicted in Figure 3. Defibrillation testing among patients undergoing initial ICD implantation declined from 71.6% in the second calendar quarter of 2010 to 36.4% in quarter 4 of 2015 (*P* < .001 for trend). Among selected subgroups of patients previously outlined, there were significant differences in the rate of DFT over time for each group (all *P* < .001 for trend and, using Cochran-Mantel-Haenszel test for interaction, *P* < .001 for all). However, the difference in the rate of DFT was most evident for patients undergoing primary (71.2% vs 74.0% in 2010) vs secondary (37.7% vs 47.3% in 2015) prevention indication for implant. The difference in the rate of DFT among patients at risk for an elevated defibrillation threshold vs those not at risk was 69.4% vs 72.1% in 2010 and 37.5% vs 39.9% in 2015, respectively, and among patients with and without structural abnormalities was 74.1% vs 71.6% in 2010 and 45.7% vs 39.2% in 2015, respectively.

**Figure 4.**
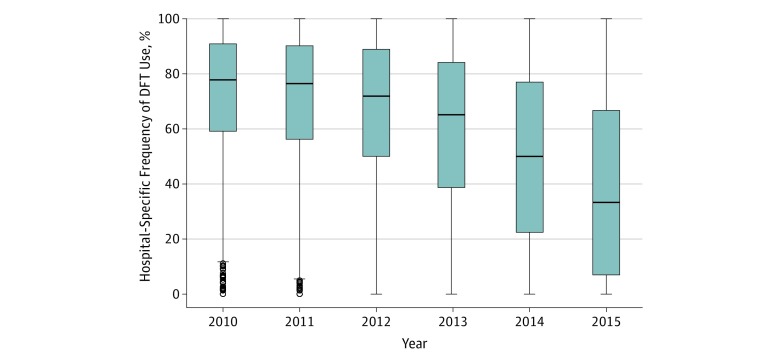
Hospital-Level Variation in Use of Defibrillation Testing The median (center line) and interquartile range (top and bottom borders of box) of defibrillation testing with median odds ratio for each study year. Whiskers bars indicate 95% CI; circles, outliers.

Hospital-level variation in performing DFT increased over time. With a decline in the median rate of DFT, there was a corresponding increase in the interquartile range (Figure 4). The interquartile range increased from 31.7% in 2010 to 59.7% in 2015. The MOR for use of DFT in the entire cohort was 3.88 (95% CI, 3.69-4.08), which increased over time from 3.78 (95% CI, 3.54-4.03) in 2010 to 6.05 (95% CI, 5.61-6.52) in 2015, indicating that in 2015 a randomly selected patient receiving DFT at a hospital with high testing rates would have a 6-fold higher odds of being tested than if he or she had received care at a hospital with a low DFT rate. Among hospitals in the fourth quartile (most use of DFT), DFT declined from 89.6% to 78.3%; the rate in hospitals in the first quartile (least use of DFT) declined from 40.3% to 9.9% (*P* < .001 for trend for each).

### Outcomes After DFT

A total of 6493 adverse events (1.3%) and 7364 adverse events or death (1.5%) occurred among patients undergoing DFT during the study period in the index hospitalization. The adjusted risk of adverse events (OR, 1.27; 95% CI, 1.21-1.34) and adverse events or death (OR, 1.31; 95% CI, 1.24-1.37) was higher in patients not receiving DFT compared with patients who underwent DFT.

## Discussion

This study evaluating the use of DFT among 499 211 patients in the NCDR ICD Registry in 1794 US hospitals has several findings. First, the use of DFT at the time of ICD implantation appeared to decline significantly between 2010 and 2015. Second, there seemed to be variation in the use of DFT based on patient, clinician, and hospital characteristics, and the omission of DFT was possibly associated with the severity of comorbidities. Third, the temporal decline in DFT appeared to be accompanied by an increase in hospital-level variation.

### Trends in DFT

Historically, DFT was performed in all patients undergoing ICD implantation. However, with improvements in technology, current-generation ICDs are capable of high-energy, biphasic, and tunable defibrillation waveforms such that successful defibrillation occurs in approximately 90% of clinical shocks.^6,10^ A post hoc analysis of the Sudden Cardiac Death in Heart Failure Trial published in 2008 demonstrated that baseline DFT did not indicate the possibility of long-term mortality or shock efficacy.^8^ The Shockless Implant Evaluation trial assessed the effect of DFT in patients undergoing initial left-sided ICD implantation.^22^ The no-testing arm was noninferior to the testing arm for the primary outcome of arrhythmic death or failed appropriate shock. A second randomized trial found that forgoing DFT was noninferior to routine DFT using a primary end point of average first-shock efficacy for ventricular tachycardia and ventricular fibrillation episodes.^23^ Released in 2015, an expert consensus statement on optimal ICD programming and testing provided a IIa recommendation to “omit defibrillation efficacy testing in patients undergoing initial left-pectoral transvenous ICD implantation procedures where appropriate sensing, pacing, and impedance values are obtained with fluoroscopically well-positioned RV [right ventricular] leads.”^24^^(p70) ^The results of the present study may provide evidence that the use of DFT was already declining by the time that these randomized clinical trials were completed and the expert consensus statement had been released. This reduction likely reflects implanting operators’ views regarding the value of DFT based on the literature as it was published.

Many questions remain unanswered regarding DFT owing to limited data to provide evidence-based recommendations. It is reasonable to withhold DFT among patients with severe heart failure, valvular disease, or atrial arrhythmias and subtherapeutic anticoagulation. Alternatively, it is reasonable to perform testing among patients with right-sided implants or those with advisory leads at the time of generator change, and it is recommended among patients undergoing subcutaneous ICD.^24,25^ Further questions remain regarding how best to approach patients whose conditions were poorly represented in trials (hypertrophic cardiomyopathy or congenital heart disease), those who are at very high risk for recurrent arrhythmias (secondary prevention indication), or those at risk for an elevated defibrillation threshold (amiodarone use, right-sided generator implants).

We found that, while DFT has declined overall, testing in certain populations varied. For instance, patients with a secondary prevention indication and structural abnormalities were more likely to undergo testing compared with those with a primary indication and those without structural abnormalities. This difference likely reflects the fact that these patients are at higher risk of appropriate ICD therapies, in which case testing is believed to provide reassurance that the system will abort ventricular arrhythmias when needed. Alternatively, the number and severity of comorbidities appeared to be related to the omission of DFT, which reflects that patients who are sick and/or frail were preferentially chosen to not undergo DFT. Prior factors that appeared to be associated with the absence of DFT include advanced age, a history of heart failure, advanced NYHA class status, atrial arrhythmias, lower ejection fraction, a wider QRS interval, and a higher serum creatinine level.^26^ However, paradoxically, these patients who are not undergoing DFT may be more likely to benefit. Further studies are needed to determine which patients gain the most benefit from DFT.

### Hospital-Level Variation in Use of DFT

We found significant hospital-level variation in the performance of DFT, which was present at the beginning of the study years and increased over time. An MOR exceeding 6 in 2015 may reflect substantial variability in DFT use. There are many plausible explanations for this variability, including the availability of anesthesia specialists to provide deep sedation, clustering of graduated fellows practicing in regions where they trained, or a monetary incentive to performing testing as there is a separate *Current Procedural Terminology* code for DFT that can be charged. In addition, the differences in regional use of DFT may reflect changes in payment structures in which reimbursement is bundled and there might be less incentive to perform additional procedures. However, given that hospitals in the fourth quartile (most use of DFT) had only an 11% change in use of DFT, while those in the first quartile had a 30% change in use of DFT over the study years, it seems that clinicians who routinely perform DFT have not changed their practice while others have markedly reduced their use of DFT.

The reasons for describing this change is that a fundamental aspect to quality improvement is reducing unwarranted variation in care. Identifying hospital-level variation is a first step in understanding the extent to which patient factors influence variation in care compared with institutional factors, which can serve to be processes for interventions in attempts to improve the quality of care.

### Outcomes After DFT

In secondary analysis, we did not find evidence that DFT was associated with harm; in fact, there was a higher risk of adverse events among patients who did not undergo DFT. However, this outcome should not be interpreted as indicating that DFT is beneficial and/or without risk, and this finding is in direct contrast to prior literature suggesting that, while rare, serious adverse events occur during DFT.^13^ The results of our study may be explained by 2 reasons: the number and severity of measured comorbidities was associated with the lack of DFT, such that patients who are sick and/or frail were preferentially chosen to not undergo DFT, and an effect-cause relationship, in which DFT was not performed because of an intraprocedural complications.

### Evolution of Practice Patterns

The evolution of medicine occurs through a process of adoption and/or deadoption of practices. More often, there is a focus on adoption of newer interventions that outperform established practices. However, there must also be deadoption of established standards, not because a better intervention has been developed, but because what was once thought to be beneficial is not. A well-recognized example of this was the previously held belief that suppression of ventricular ectopy following acute myocardial infarction, which was an independent predictor of mortality, would improve arrhythmic death. However, the use of class 1C drugs, which successfully reduced ventricular ectopy, also resulted in an excess of mortality; thus, there was a deadoption of the use of class 1C drugs among patients with structural heart disease.^27^ There are numerous other examples of deadoption, which are best seen in the evolving changes in clinical guidelines.^28,29^ The present study may provide a perspective on the pace of deadoption of a once-held belief that DFT needs to be performed during ICD implantation. Despite mounting evidence that omission of DFT is safe, there is still significant variation in its use, with some institutions continuing to apply DFT frequently, which is independent of patient characteristics. As with adoption, deadoption requires a focus on the cultural aspects of practice, which have evolved from not only evidence-based practices, but also personal experiences, conflicts, and biases.

### Limitations

This study has limitations. Certain factors should be considered in the interpretation of the findings. First, the reasons behind decisions to perform or not perform DFT are not gathered from the NCDR ICD Registry. Second, the decision on use of DFT in a small portion of patients was based on their upper limit of vulnerability, which does not necessarily have the same risks as the lowest energy required (ie, patients do not need to have induction of ventricular fibrillation during the upper limit of vulnerability). In addition, defibrillation threshold testing is likely a misnomer for many centers in which a single shock of 10 to 15 J or greater is an acceptable defibrillation safety margin. Third, high-risk features exist for an elevated defibrillation threshold, including right-sided generator implants, which are not recorded in the NCDR ICD Registry. In addition, more contemporary data beyond 2015 would be insightful to describe the patterns in use of DFT, particularly because both randomized clinical trials and the expert consensus statement were published at the end of the time of data acquisition; however, at the time of our study, this information was not available for analysis owing to changes in the NCDR ICD Registry data collection form.

## Conclusions

There appears to have been a significant reduction in the use of DFT for initial ICD implantation in US hospitals over time, declining from 72% in the first quarter of 2010 to 36% in the last quarter of 2015. Yet, although the overall use of DFT has declined, there has been an increase in the institutional variability in performing DFT with an increase in the MOR from 3.78 in 2010 to 6.05 in 2015. This variability in deadoption of DFT could reflect differences in practice culture despite mounting evidence and guidance that DFT may not be necessary at the time of ICD implantation.
